# The evaluation of risk factors for recurrent hospitalizations resulting from wheezing attacks in preschool children

**DOI:** 10.1186/s13052-015-0201-z

**Published:** 2015-11-17

**Authors:** Sebnem Ozdogan, Burcu Tabakci, Ayse Sirin Demirel, Bilge Atli, Gulser Esen Besli, Gulsen Kose

**Affiliations:** Sisli Hamidiye Etfal Research and Training Hospital, Istanbul, Turkey; Sinan Ercan Sok, Isiklar 1 Apt. No 34, Da: 23, 34742, Kozyataği, Istanbul, Turkey; Istanbul Medeniyet University, Faculty of Medicine, Goztepe, Turkey

## Abstract

**Background:**

We aimed to evaluate the risk factors in preschool children admitted to inpatient services with a diagnosis of recurrent attacks of wheezing.

**Method:**

The medical files of 44 preschool children with 2 or more recurrent hospitalizations resulting from wheezing between November 2011 and January 2012 were retrospectively investigated.

**Results:**

There were 28 males (64 %) and 16 females. The median age was 14 months (2.0–50). The median numbers of previous wheezing attacks and hospitalizations were 4 (2–10) and 2 (2–8), respectively. Fourteen patients (32 %) had been treated for gastroesophageal reflux (GER). The previous and recent hospital evaluations were investigated. Bronchopulmonary dysplasia and anemia were significantly more common in patients with 3 or more hospitalizations for wheezing than in those with 2 hospitalizations (*p* = 0.010 and *p* < 0.001, respectively). A review of the cases with 3 or more hospitalizations revealed that a history of GER and anemia were significant risk factors.

**Conclusion:**

Anemia and GER are risk factors for recurrent hospitalizations resulting from wheezing and should be treated. If the history and physical examination suggest asthma, inhaler therapy treatment should be administered, with other investigations planned for patients who do not respond to treatment as expected.

## Background

Recurrent wheezing attacks occurring during the pre-school period represent a significant healthcare concern and comprise one of the most common causes of emergency department visits and hospitalizations. The Children’s Respiratory Study (CRS) in Tucson reported a wheezing prevalence of 32 % during the first year of life, which decreased to 17.3 % at 2 years of age and to 12 % at 3 years of age [1]. One in three children experience at least one acute wheezing attack before the age of three years [2].

Recurrent wheezing impairs the quality of life for the patient and his/her family and causes a significant economic burden resulting from the healthcare expenses associated with the condition [3–5]. A phone call-based study by Bisgaard et al. detected the prevalence of recurrent wheezing among children between 1 and 5 years of age as 27 %, 29 % and 48 % in America, Northern Europe and Southern Europe, respectively. The rate of presentation to emergency departments and hospitalization was reported to be 16 % and 12 %, respectively, within the last 6 months [6].

A multinational study conducted in 17 centers in Latin America and Europe reported a wheezing ratio of 45.2 % and a recurrent wheezing prevalence of 20.3 % during the first year of life. The same study reported a rate of emergency department visits for wheezing of 71.1 % and a rate of hospitalization for recurrent wheezing of 26.8 % [7].

Whereas the most common cause of recurrent wheezing is asthma in all age groups, gastroesophageal reflux disease (GER), foreign body aspiration, bronchopulmonary dysplasia (BPD), bronchiolitis obliterans, an immunodeficiency, primary ciliary dyskinesia, vocal cord dysfunction, cardiac etiologies and structural abnormalities should be considered in the differential diagnosis [8]. A definitive diagnosis is based on invasive investigations such as radiological studies, laboratory values and bronchoscopy as well as the clinical history and physical examination. Because of the lack of consensus on the treatment for recurrent wheezing, many investigations could be conducted, and various treatments could be administered.

We evaluated the medical records of preschool children with a history of at least two hospitalizations resulting from attacks of wheezing.

## Methods

The medical files of 44 children with two or more recurrent hospitalizations resulting from wheezing were retrospectively examined. The study was conducted in the Pediatric Inpatient Service at Sisli Hamidiye Etfal Training and Research Hospital between November 2011 and January 2012. We included children between 1 month and 5 years of age with complete records of at least 2 hospitalizations for wheezing. Children younger than 1 month and older than 5 years of age and those with incomplete or missing records were excluded. The demographics, previously requested investigations and current hospitalization records were investigated, as were the treatment approaches. The ethics committee of the hospital granted approval for the study.

### Investigations

In patients with recurrent hospitalizations for wheezing, laboratory tests, imaging studies and, in some cases, bronchoscopy, were performed to aid the differential diagnosis. Detailed explanations of each of the tests performed in this study follow.

### Blood tests

A hemogram, the total IgE values, specific IgE-inhalant allergens, specific IgE-food mixtures, and serum immunoglobulin count were obtained. For specific IgE-inhalant allergens, tree pollens, betula berrucosa, meadow pollens, weed pollens, house dust, dermatophagoides pteronyssinus, dermatophagoides farinae, fungi, yeast, mold and animal epithelia were investigated. For food mixtures, egg white, milk, fish (gadiformes), flour, peanut and soybeans were investigated. Anemia is defined as a hemoglobin level of less than the 5th percentile for age. The serum total IgE levels were determined to be normal or high based on the participants’ ages. Eosinophilia refers to an absolute eosinophil count in the peripheral blood of ≥ 500 eosinophils/microL/Specific IgE inhalant allergens and food allergens were determined to be normal or high, based on the reference values.

### Radiological investigations

Chest radiography and, in selected patients, computed thoracic tomography were performed. The investigation results were assessed by a radiologist.

### Purified protein derivative skin test

Purified protein derivative (PPD) skin testing was performed in the patients with a history of tuberculosis contact. The PPD solution was obtained from the Tuberculosis Dispensary in prefilled injectors and injected under appropriate conditions into the forearm of the patients via an intradermal route; the diameter of the induration occurring after 72 h was measured. For the patients without risk factors, an induration ≥ 15 mm was considered positive.

### Sweat test

The sweat test was performed using the quantitative pilocarpine iontophoresis method in accordance with the international standards.

### Esophageal pH monitoring

The patients underwent 24-h lower esophageal pH monitoring for longer than 18 h (ComforTec Plus Single Use Ph Prob, Sandhill Scientific, ZEPHR, USA). If the patient were receiving anti-reflux treatment, the drugs were discontinued 7 days before the investigation. The esophageal pH monitoring test results were considered to be abnormal when the time of pH ≤ 4.0 was greater than 6 % of the duration of the pH monitoring study for children over 1 year old and greater than 10 % for children younger than 1 year of age.

### Echocardiography

Echocardiography (ECHO) was performed to assess the internal structure and functions of the heart via sound waves. A pediatric cardiologist assessed the results.

### Chest computed tomography

Chest computed tomography (CT) was performed in selected patients to provide a detailed anatomy of the mediastinum, large airways, and lung parenchyma.

### Flexible fiberoptic bronchoscopy

This investigation was performed by a pediatric pulmonologist under general operation room conditions using general or local anesthesia.

### Statistical analysis

The descriptive properties of the variables were detected (the mean, median and frequency). The compliance of the variables with the normal distribution was checked. The Student’s t-test was used for the comparison of the variables with normal distribution. A comparison of the categorical variables was made using the chi-square test. Chi square testing (with the Fisher exact test where indicated by low expected cell counts) was used for this purpose. A univariate analysis was performed in the logistic regression analyses. The variables, which were *p* < 0.250 and considered potentially clinically significant, were included in the multivariate analysis, and a multiple logistic regression analysis was performed; *p* < 0.05 was considered significant. The Statistical Package for Social Sciences (SPSS) software, v. 17, (Chicago, USA) was used to evaluate the results.

## Results

A total of 52 patients aged 1 month to 5 years with at least 2 recurrent hospitalizations resulting from wheezing were admitted during the study period. A total of 44 patients (16 females, 28 males) with complete medical records were enrolled in this study. The clinical characteristics of the patients are presented in Table 1. The median age was 14 months (2.0–50). Two patients (5 %) were below the 3^rd^ percentile for height and weight. The median number of previous wheezing attacks and hospitalizations were 4 (2–10) and 2 [2–8], respectively. Eight of the patients were born prematurely (18 %), 14 (32 %) were admitted to the NICU, 7 (16 %) were intubated and 5 (11 %) were diagnosed with BPD. Fifteen patients (34 %) were breastfed for less than 6 months, and 25 patients (57 %) were bottle-fed at night. Fourteen patients (32 %) had been treated for GER. Twenty-one patients (48 %) had a parent or sibling with a history of asthma, allergic rhinitis or eczema, and 29 patients (66 %) had been exposed to smoke. The median number of people living in the same house as the patient was 4 (3–12). A review of the treatment regimens revealed that 9 patients (21 %) were treatment-naïve (with nebules), and only 10 had received nebule treatment. Fifteen patients used salbutamol syrup as prescribed.

**Table 1:** Clinical characteristics of patients, illustrating risk factors for recurrent wheezing

		Median	Range
Age (months)		14	2.0–50
Number of recurrent wheezing attacks		4	2–10
Hospital admissions for recurrent wheezing		2	2–8
Number of people living in the same house		4	3–12
		n	%
Sex (males)		28	64
Height and weight <3 %		2	5
Birth history	Prematurity	8	18
	BPD^*^	5	11
	NICU admission^+^	14	32
	History of intubation	7	16
Breastfeeding <6 months		15	34
Nocturnal feeding		25	57
GER history		14	32
Inhaler use	Regular	10	23
	naive	9	21
Salbutamol syrup		15	34
Smoking exposure		29	66
Parental asthma/AR/eczema		21	48

^*^Bronchopulmonary dysplasia, ^+^Neonatal intensive care unit

The previous and recent hospital evaluations were investigated. The laboratory results are summarized in Table 2. Based on the hemograms obtained during the most recent hospitalization, 15 patients (34 %) were anemic, and 6 (14 %) had eosinophilia. Pulmonary radiography had been performed in all of the patients, and 23 (52 %) patients had infiltration/atelectasis. Immunoglobulins, including IgA, IgG and IgM were obtained in 38 (86 %) of the patients, and the levels were reported to be within the normal limits by age. Esophageal pH monitoring had been performed in 10 (23 %) patients, and 5 patients (50 %) were reported to have reflux. Two of the 30 patients who underwent echocardiography (7 %) were reported to have abnormal results. The immunoglobulin E level had been obtained in 39 (88 %) patients, 21(53 %) of whom had a high immunoglobulin E level. The inhalant panel was obtained for 31 patients and found to be positive for 3 patients (10 %). A PPD test was applied in 17 patients (39 %) with a positive tuberculosis contact history, and the results were negative. Ten patients (23 %) had undergone a sweat test, and the results for these patients were normal. Five of the patients undergoing chest CT (11 %) were reported to have infiltration. Three patients (7 %) had permanent wheezing and were non-responsive to nebule treatment; these patients underwent bronchoscopy, and no pathology, other than increased secretion, was reported.

**Table 2:** Summary of the results of investigations performed

Test	Subjects tested (%)	Abnormal results (%)
Hemogram	44/44 (100)	15/44 (34)–anemia
Eosinophilia	44/44 (100)	6/44 (14)
CXR	44/44 (100)	23/44 (52)–infiltration/atelectasis
IgA, IgG, IgM	38/44 (86)	0/38 (0)–below normal
IgE	39/44 (88)	21/39 (53)–high
Esophageal pH monitoring	10/44 (23)	5/10 (50)–findings consistent with reflux
Inhalant/food panel	31/44 (70)	3/31 (10)–positive
ECHO	30/44 (68)	2/30 (7)–abnormal
ppd	17/44 (38)	0/17 (0)–positive
Sweat test	10/44 (22)	0/10 (0)–positive
Chest CT	5/44 (11)	5/5 (100)–infiltration
FOB	3/44 (7)	3/3 (100)–increased secretions

*CXR* Chest X ray, *Ig* Immunglobulin, *ECHO* Echocardiography, *ppd* Purified protein derivative skin test, *CT* Computerized tomography, *FOB* Flexible fiberoptic bronchoscopy

The patients were divided into 2 groups as patients with 2 and patients with 3 or more hospitalizations. The risk factors and investigation results were compared (Table 3). The patients with 3 or more hospitalizations resulting from wheezing had a statistically significantly higher rate of BPD and anemia relative to the other group (*p* = 0.01 and *p* < 0.001, respectively).

**Table 3:** Comparison of the patients with 2 and patients with 3 or more hospitalizations due to recurrent wheezing attacks

	2	3 or more	
	n: 24	n: 20	
	n (%)	n (%)	*p*
Gender (male)	14 (58)	15 (%75)	^*^0.34
Age(months), mean	19.7 ± 14.3	17.0 ± 10.6	^^^0.48
History of NICU^a^ admission	5 (21)	9 (45)	^*^0.16
History of intubation	2 (8)	5 (25)	^*^0.13
BPD^b^	0 (0)	5 (25)	^*^0.01^**^
GER^c^	5 (21)	9 (45)	^*^0.16
Preterm delivery	3 (13)	5 (25)	^*^0.29
Nocturnal feeding	14 (58)	11 (55)	^*^1.00
Nocturnal feeding	14 (58)	11 (55)	^*^1.00
Pathologic pH monitoring	2 (8)	3 (15)	^*^0.34
IgE level (high)	10 (42)	8 (40)	^*^0.57
Abnormal CXR^d^	13 (54)	10 (50)	^*^1.00
Abnormal Chest CT^e^	1 (4)	4 (20)	^*^0.16
Positive inhalant/food panel	1 (4)	2 (10)	^*^0.77
Eosinophilia	3 (13)	3 (15)	^*^1.00
Anemia	3 (13)	12 (60)	^*^ < 0.00^**^
Parenteral asthma/eczema/AR^f^	8 (33)	5 (25)	^*^0.78
ICS^g^ use	16 (67)	14 (70)	^*^1.00
Exposure to smoking	15 (63)	14 (70)	^*^0.83

Neonatal intensive care unit^a^_,_ Bronchopulmonary dysplasia^b^, Gastroesophageal reflux^c^ , Chest Xray^d^, Computed tomography^e^, Allergic rhinitis^f^, Inhaler corticosteroid^’g^

^^^Results expressed as mean ± SD (student’s t-test). Chi-square test or Fisher’s exact test. ^**^Statistically significant

Table 4 reveals the risk factors of the patients with 3 or more hospitalizations. The multivariate regression analysis revealed that the odds ratio for hospitalization resulting from a history of GER and the presence of anemia is 6.29 and 17.03, respectively.

**Table 4:** Multivariate logistic regression analysis showing the risk factors for 3 or more hospitalization for wheezing attacks

	Adjusted OR	CI 95 %	*p*
Anemia	17.02	3.03–95.44	0.00
GER	6.28	1.99–33.33	0.03

*CI* Confidence interval, *OR* Odds ratio, *GER* Gastroesophageal reflux

## Discussion

Because of the lack of consensus on the description, assessment and treatment of recurrent wheezing in young children, these cases are quite difficult for clinicians to manage. Many unnecessary investigations are conducted, and inadequate treatment is provided. We investigated the demographics, the requested investigations, and the results for pre-school children with recurrent hospitalizations resulting from wheezing. A comparison of the patients with 2 and the patients with 3 or more hospitalizations resulting from wheezing revealed that the patients with 3 or more hospitalizations had a statistically significantly higher rate of BPD and anemia relative to the other group. The regression analysis revealed that a GER history and presence of anemia were significant risk factors in patients with 3 or more hospitalizations resulting from wheezing.

Several studies have reported that boys have an increased risk of early persistent wheezing and allergic sensitization [9, 10]. In our study, 64 % of the patients with recurrent wheezing attacks were boys.

Breastfeeding protects against respiratory infections during the early period of life. However, the relationship between breastfeeding and wheezing has not been elucidated [11]. Whereas there are clinical studies suggesting that breastfeeding is protective against wheezing, other studies have not detected an association. We failed to detect an association between breastfeeding and recurrent wheezing, although 34 % of the patients were breastfed for less than 6 months, which is most likely because of the small sample size.

Passive smoking has a strong correlation with respiratory complaints [12]. In this study, we detected a rate of passive smoking as high as 66 %; however, we failed to show passive smoking as a risk factor in patients with 3 or more hospitalizations resulting from wheezing. In the British Cohort Study, a strong correlation was demonstrated between maternal smoking and the presence of wheezing during early childhood; however, such a correlation could not be shown for the subsequent period [13]. The Tucson CRS study suggested that maternal prenatal smoking affects in utero lung function and increases the risk of wheezing during the first 3 years of life [14]. A recent mother and child cohort study in Norway reported that smoking by the grandmother while pregnant with the mother increases the risk of asthma in the grandchild, independent of the maternal smoking status [15]. In this study, we did not investigate the history of smoking during pregnancy.

A review of the cases with 3 or more hospitalizations for wheezing revealed that the presence of anemia increased the risk of hospitalization 17.02 fold. The studies investigating the association between anemia and lower respiratory infections are limited. Iron deficiency anemia affects the immune response and alters the metabolism of pathogens. A low tissue hemoglobin level impairs tissue oxygenation and represents a risk for lower respiratory infections in children [16]. The literature data suggest that the presence of anemia increases the risk of acute lower respiratory infection 3 to 6 fold [16, 17]. In a randomized, controlled study in Sri Lanka, iron supplementation reduced morbidity in children with and without an upper respiratory tract infection [18]. There are studies suggesting that maternal anemia during pregnancy is a risk factor for recurrent wheezing [19]. Maternal anemia was not investigated in our study.

Reviewing the patients with recurrent hospitalizations resulting from wheezing, we observed that the presence of GER increased the risk of 3 or more hospitalizations 6.29 fold. In our study, 14 patients had been on GER treatment, and 5 of the 14 had abnormal pH monitoring results. The presence and treatment of GER has a significant role in recurrent wheezing. A systematic review of the clinical studies conducted between 1966 and 2008 revealed a mean GER prevalence of 20 % (ranging from 19.3 to 80 %) in children with asthma [20]. A study investigating the association between the recurrence of respiratory symptoms and GER detected GER in 35 % of the patients, which included 40 % of the enrolled cases of reactive airway disease. In the same study, the respiratory complaints had started below the age of one in 86 % of the patients with GER. A marked reduction in wheezing was observed 3–6 months after anti-reflux treatment [21]. Empiric GER treatment would be an appropriate approach in patients with recurrent hospitalizations resulting from wheezing and in cases of no response or an inadequate response to ICS treatment.

Patra et al. found that 42 % of the patients below the age of 1 with wheezing had positive GER investigations. They report that GER is a significant cause of recurrent wheezing in patients below 2 years of age and recommend GER investigations in patients with severe attacks having an onset below the age of 1 year [22]. A trial by Sheikh et al. detected silent chronic aspiration secondary to difficulty of swallowing without GER in 13 infants with chronic respiratory symptoms [23].

Consensus was not achieved on the description, assessment and treatment of wheezing in pre-school children; however, a trial of ICS and montelukast treatment in patients with recurrent wheezing, with discontinuation of treatment in non-responders, was recommended [24].

In an observational study by Saglani et al., invasive tests, including computed chest tomography, blood tests, a nasal cilia biopsy, flexible bronchoscopy with bronchoalveolar lavage, and pH monitoring, were performed in 47 patients, between 3 months and 5 years of age, who experienced recurrent wheezing despite ICS treatment. One-third of the patients had abnormal investigation results, and these patients were diagnosed with an airway abnormality, GER, eosinophilic airway inflammation, and bacterial infection [25]. In our study, 80 % of the patients had not received previous ICS treatment or were not using the therapy regularly.

Regardless of the age at onset, atopic findings, triggering factors or the frequency of wheezing, the most common cause of recurrent wheezing is asthma [26]. A study in Finland suggested that, based on a 6-month follow-up, more than one-third of children below 2 years of age, hospitalized for wheezing, were diagnosed with asthma, and the risk was high, particularly in those with recurrent wheezing and allergic findings [27].

Salbutamol syrup remains in use for the treatment of wheezing. Whereas the Global Initiative for Asthma (GINA) and National Asthma Education and Prevention Program (NAEPP) consensus reports do not include salbutamol syrup in the treatment protocol for asthma for patients below 5 years of age, the syrup is frequently used in the treatment of wheezing [28–31]. Whereas salbutamol syrup should not be used because of the absence of efficacy for the treatment of wheezing and the presence of side effects, this treatment remains in use because of economic reasons and the societal prejudice against inhaled treatments [32]. Physicians have a great role in the implantation of treatment protocols through efforts to inform society and establishing consensus reports.

Considering that the study was conducted in winter and that one-half of the pulmonary radiography investigations were reported as increased ventilation and infiltration/atelectasis, the most likely cause of wheezing was infection. We were unable to perform a respiratory viral panel. There were 14 patients with GER findings (31.8 %), and 5 patients (11.4 %) had esophageal pH monitoring results consistent with reflux. The sensitivity, specificity and clinical utility of pH monitoring for extra esophageal indications are not well established.

The high serum IgE level, eosinophilia, inhalant/food panel detected in 53 %, 14 %, and 10 % of the patients, respectively, suggest the presence of changes secondary to infection rather than an atopic background. An increased serum IgE level might be associated with an acute viral infection in atopic and non-atopic children [33]. IgE production is secondary to an inflammatory process occurring in the asthmatic airway rather than to a specific allergen [34]. The presence of eosinophilia might be explained by parasitosis as well as atopy. Of the other tests requested, the immunoglobulin panel, PPD, sweat test and ECHO investigation yielded normal results in a larger portion of patients. The low frequency of the diagnoses of immunodeficiency, cystic fibrosis and cardiac abnormality that should be considered in the differential diagnosis of recurrent wheezing might be attributed to the small number of patients. If the number of the patients were larger, we could hypothesize that the probability of diagnosing the above conditions would increase.

The strength of our study is that it is the first to assess the risk factors for 2 or more recurrent hospitalizations resulting from wheezing. An important limitation of this retrospective study results from missing data, incomplete data, unknown confounders, and the small sample size.

## Conclusion

Anemia and GER are risk factors for recurrent hospitalizations for wheezing and should be treated. In cases of recurrent wheezing, the investigations to be performed are quite limited if the history and the physical examination are not atypical. Various non-invasive and invasive investigations are increasingly performed to rule out other potential pathologies and as a measure against malpractice claims. Most of these investigations are unnecessary and expensive and lead to radiation and trauma to children. If the physical examination and history are not abnormal, an ICS trial should be administered, with further investigations planned for non-responders; this protocol would be more appropriate than requesting investigations to rule out potential diagnoses.
